# Supplementary material for: Computational identification and experimental characterization of preferred downstream positions in human core promoters
Source: PLoS Comput Biol. 2021 Aug 12;17(8):e1009256. doi: 10.1371/journal.pcbi.1009256 (PMC8384218; doi:10.1371/journal.pcbi.1009256)
Supplement: S1 Table — (DOCX) [file pcbi.1009256.s003.docx]

**S1 Table.** **Three bp periodic distributions of ATG in human promoter classes 4-6.**

| **Position** | **Class 4** | **Class 5** | **Class 6** |
| --- | --- | --- | --- |
| +9 | 0 | 4 | 8 |
| +10 | 12 | 0 | 4 |
| +11 | 3 | 5 | 0 |
| +12 | 0 | 1 | 5 |
| +13 | 13 | 1 | 0 |
| +14 | 5 | 8 | 0 |
| +15 | 0 | 3 | 10 |
| +16 | 9 | 0 | 6 |
| +17 | 3 | 8 | 0 |
| +9, +12, +15 | 0 | 8 | 23 |
| +10, +13, +16 | 34 | 1 | 10 |
| +11. +14, +17 | 11 | 21 | 0 |
