# Supplementary material for: Computational identification and experimental characterization of preferred downstream positions in human core promoters
Source: PLoS Comput Biol. 2021 Aug 12;17(8):e1009256. doi: 10.1371/journal.pcbi.1009256 (PMC8384218; doi:10.1371/journal.pcbi.1009256)
Supplement: S2 Table — (DOCX) [file pcbi.1009256.s004.docx]

**S2 Table.**  **Distribution of promoter classes with TATA-box status and “shape” status (narrow/broad)**

*Drosophila melanogaster*

| **Classes** | **All** | **TATA+** | **TATA−** | **Narrow** | **Broad** |
| --- | --- | --- | --- | --- | --- |
| All | 16970 = 100% | 2598 = 15.3% | 14372 = 84.7% | 5968 = 35.2% | 11002 = 64.8% |
| Class 1: | 6397 = 100% | 750 = 11.7% | 5647 = 88.3% | 3850 = 60.2% | 2547 = 39.8% |
| Class 2 | 5710 = 100% | 827 = 14.5% | 4883 = 85.5% | 1006 = 17.6% | 4704 = 82.4% |
| Class 3: | 4863 = 100% | 1021 = 21.0% | 3842 = 79.0% | 1112 = 22.9% | 3751 = 77.1% |

*Homo sapiens*

| **Classes** | **All** | **TATA+** | **TATA−** | **Narrow** | **Broad** |
| --- | --- | --- | --- | --- | --- |
| All | 27093 = 100% | 2823 =10.4 % | 24270 = 89.6% | 3206 = 11.8% | 23887 = 88.2% |
| Class 1: | 24477 = 100% | 2661 = 10.9% | 21861 = 89.1% | 2972 = 12.1% | 21505 = 87.9% |
| Class 2 | 976 = 100% | 75 = 7.7% | 901 = 92.3% | 104 = 10.7% | 872 = 89.3% |
| Class 3: | 636 = 100% | 41 = 6.4% | 595= 93.6% | 68 = 10.7% | 568 = 89.3% |
| Source data for promoter classification | | ftp://ccg.epfl.ch/epdnew/D_melanogaster/005/db/promoter_coordinate.txt  ftp://ccg.epfl.ch/epdnew/D_melanogaster/005/db/promoter_motifs.txt  ftp://ccg.epfl.ch/epdnew/H_sapiens/005/db/promoter_coordinate.txt  ftp://ccg.epfl.ch/epdnew/H_sapiens/005/db/promoter_motifs.txt | | | |

TATA-box status of each promoter were retrieved from EPD (see url accessions within the Table). The promoter class “narrow” comprises the EPD promoter types “single” and “multiple”; the class broad comprises the type “regions”.
