# Supplementary material for: Computational identification and experimental characterization of preferred downstream positions in human core promoters
Source: PLoS Comput Biol. 2021 Aug 12;17(8):e1009256. doi: 10.1371/journal.pcbi.1009256 (PMC8384218; doi:10.1371/journal.pcbi.1009256)
Supplement: S3 Table — (DOCX) [file pcbi.1009256.s005.docx]

**S3 Table. The EM algorithm (this study) makes similar predictions as the SVRb model (57).**

| Human promoter | Sequence (+17 to +35) | Class 3 (EM)  log score | SVRb  score |
| --- | --- | --- | --- |
| LRCH4 | CCGCCGGGAGCGGATGGCG | 5.19 | 6.15 |
| LRCH4_mPDP | CCGCCGGTAGCTTATGGCG | 0.61 | 0.98 |
| LRCH4_mGG | CCGCCGGGAGCGGATGGCT | 5.20 | 7.16 |
| LRCH4_pos10_m2 | GCCGGGAGCGGATGGCGGC | -4.55 | 1.19 |
| LRCH4_pos10_p2 | AGCCGCCGGGAGCGGATGG | -0.79 | 0.99 |
| LRCH4_pos18_m2 | CCCGGGAGCGGATGGCGGC | -4.24 | 0.55 |
| LRCH4_pos18_p2 | CCTCGCCGGGAGCGGATGG | -0.16 | 0.12 |
| CKS2 | TTGCCTGGGCTGGACGTGG | 2.95 | 7.97 |
| CKS2_mPDP | TTGCCTGTGCTTTACGTGG | -1.63 | 1.52 |
| CKS2_mGG | TTGCCTGGGCTGGACGTTT | 2.96 | 8.83 |
| CKS2_pos10_m2 | GCCTGGGCTGGACGTGGTT | -2.41 | 1.69 |
| CKS2_pos10_p2 | TGTTGCCTGGGCTGGACGT | -8.95 | 0.67 |
| CKS2_pos18_m2 | TCCTGGGCTGGACGTGGTT | -2.26 | 1.38 |
| CKS2_pos18_p2 | TTTCGCCTGGGCTGGACGT | -8.47 | 1.23 |
| ANP32E | TTGAAGGGGAAGGAACTGC | 5.51 | 12.89 |
| ANP32E_mPDP | TTGAAGGTGAATTAACTGC | 0.94 | 3.17 |
| ANP32E_mGG | TTGAAGGGGAAGGAACTGC | 5.51 | 12.89 |
| ANP32E_pos10_m2 | GAAGGGGAAGGAACTGCGG | -0.57 | 2.03 |
| ANP32E_pos10_p2 | CATTGAAGGGGAAGGAACT | -6.15 | 0.77 |
| ANP32E_pos18_m2 | TAAGGGGAAGGAACTGCGG | -0.43 | 2.08 |
| ANP32E_pos18_p2 | TTTCGAAGGGGAAGGAACT | -5.85 | 0.39 |
| CELF1 | CAGCGGCGGCGGGACGCGG | 2.81 | 5.44 |
| CELF1_mPDP | CAGCGGCTGCGTTACGCGG | -1.76 | 1.56 |
| CELF1_mGG | CAGCGGCGGCGGGACGCGG | 2.81 | 5.44 |
| CELF1_pos10_m2 | GCGGCGGCGGGACGCGGAG | -1.52 | 0.58 |
| CELF1_pos10_p2 | GGCAGCGGCGGCGGGACGC | -6.16 | 1.16 |
| CELF1_pos18_m2 | CCGGCGGCGGGACGCGGAG | -1.21 | 0.26 |
| CELF1_pos18_p2 | CATCGCGGCGGCGGGACGC | -6.33 | 0.93 |
| CTSA | CTGGAGAGCAAGGACGCGG | 3.81 | 8.45 |
| CTSA_mPDP | CTGGAGATCAATTACGCGG | -0.77 | 1.36 |

Columns 3 (EM algorithm, Class 3 log score) and 4 (SVRb**)** correlate with a coefficient of about 0.78.
