# Supplementary figures and images for: Computational identification and experimental characterization of preferred downstream positions in human core promoters
Source: PLoS Comput Biol. 2021 Aug 12;17(8):e1009256. doi: 10.1371/journal.pcbi.1009256 (PMC8384218; doi:10.1371/journal.pcbi.1009256)

*D. melanogaster*

— DPE  
— Shuffle

*H. sapiens*

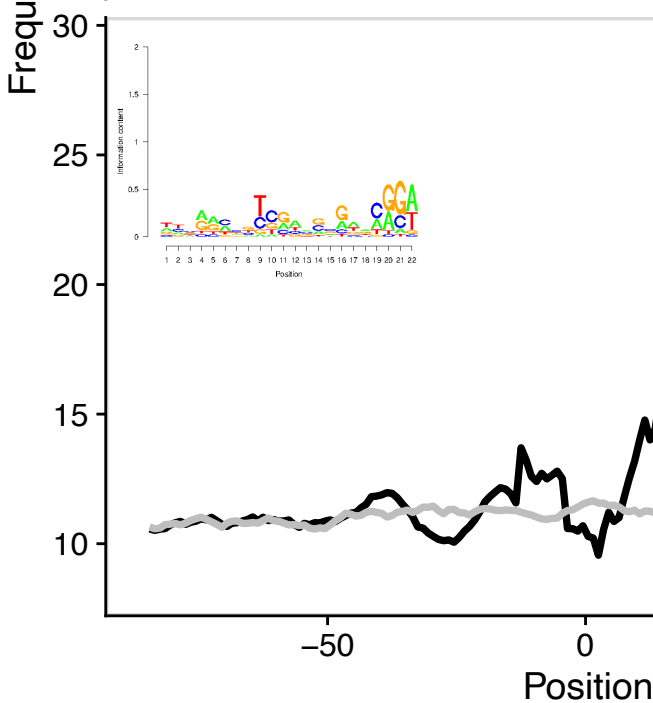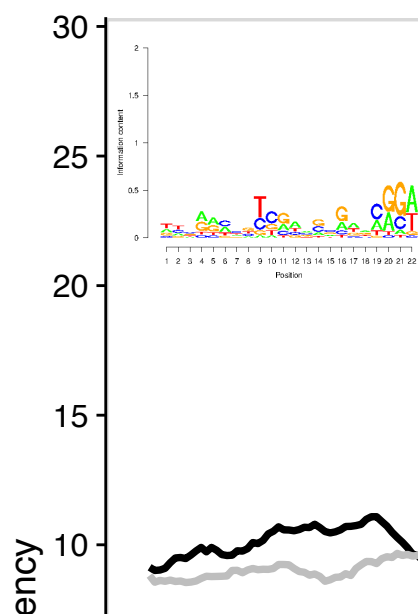

Supplement: S1 Fig — DPE motif distribution around all D. melanogaster and Homo sapiens promoters was calculated using the DPE logo depicted in the top left corner. The "shuffle" distribution is derived by scanning shuffled sequences with the DPE motif and should be considered as a background signal. Numerical data (provided in S2 File) were generated using the web interface of the OProf program from the Signal Search Analysis server [70]. (PDF) [file pcbi.1009256.s006.pdf]

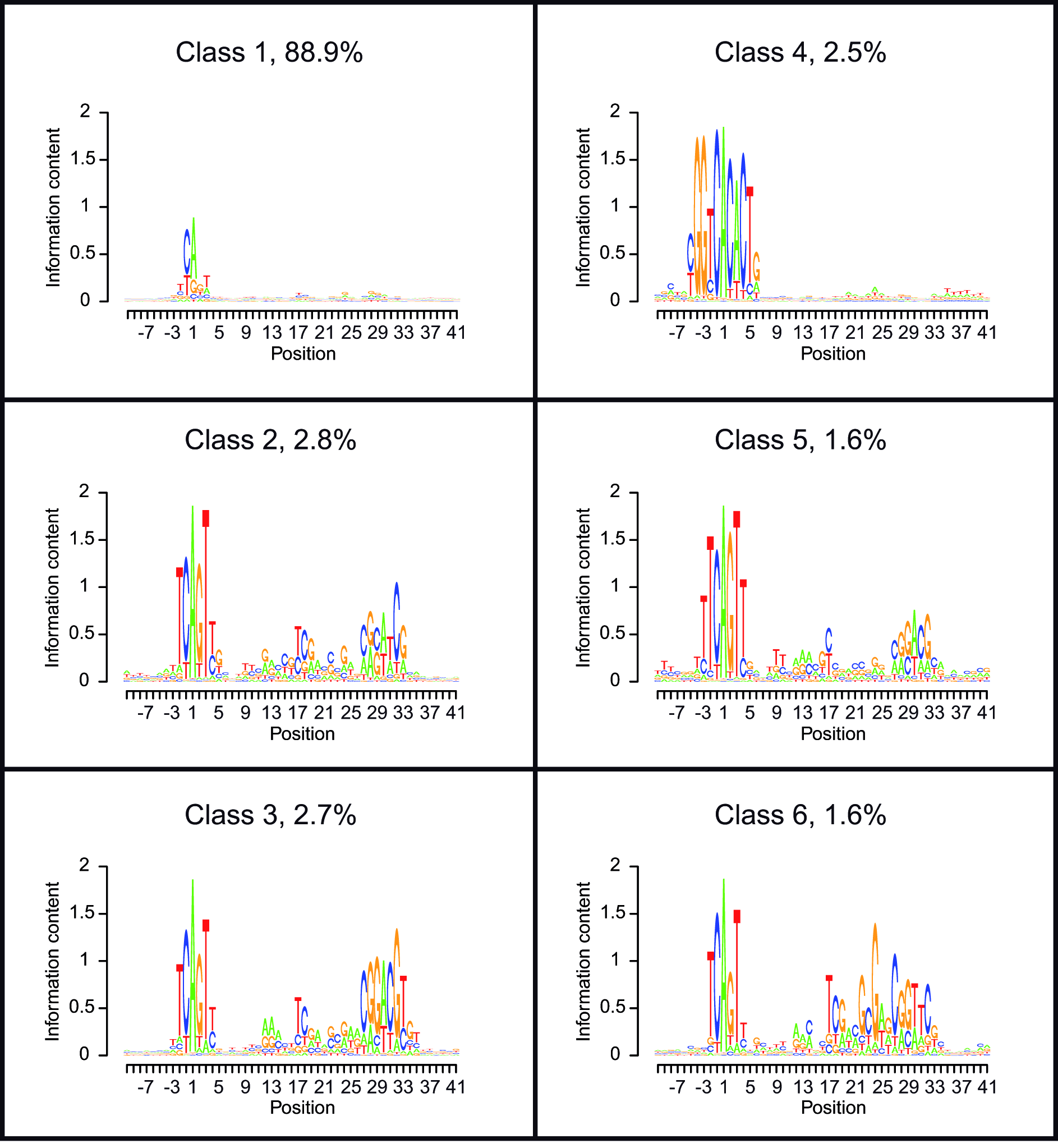

Supplement: S2 Fig — Numerical data are provided in S2 File. (TIF) [file pcbi.1009256.s007.tif]

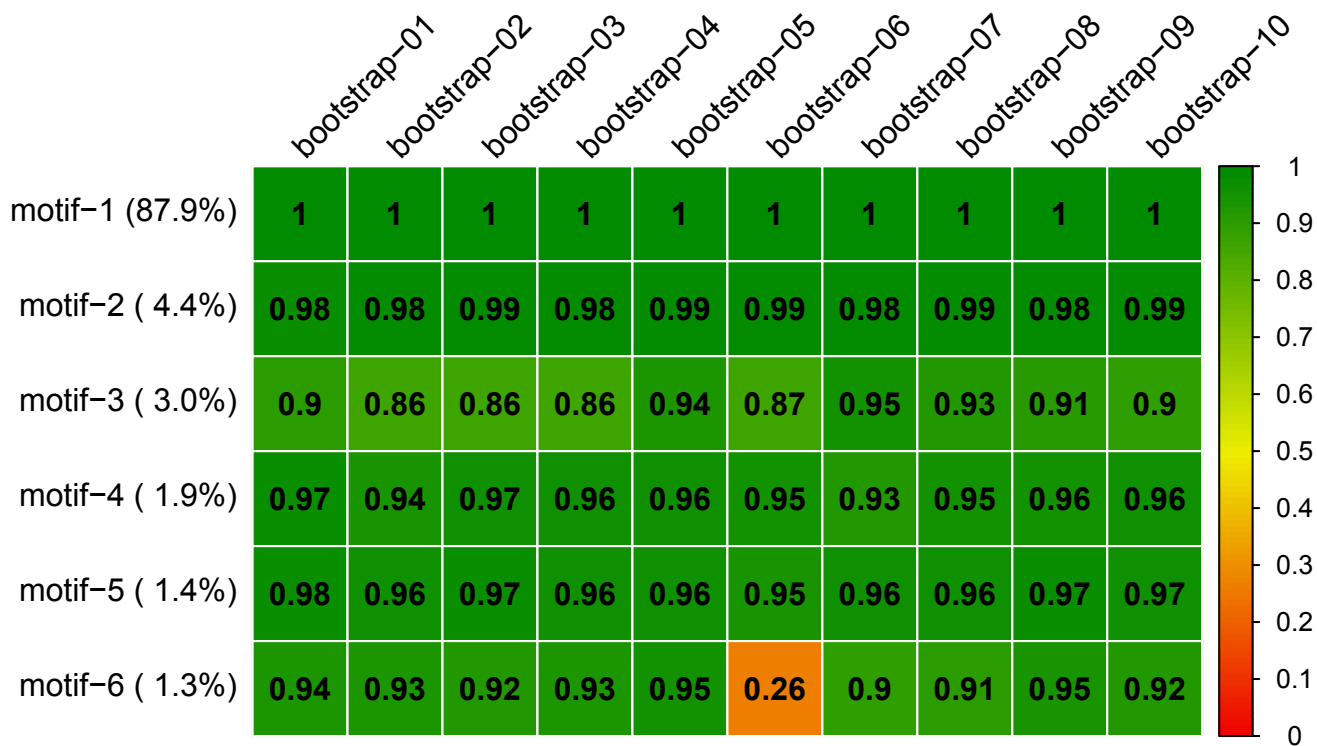

Supplement: S3 Fig — The complete promoter sequence collection was resampled 10 times. The extended partitioning algorithm was applied to the bootstrapped data sets retaining the 10 most frequently found classes. The heatmap reflects the similarity (expressed as Pearson correlation coefficients) of the newly identified motifs with the corresponding most similar motifs found in each bootstrapping round. (PDF) [file pcbi.1009256.s008.pdf]

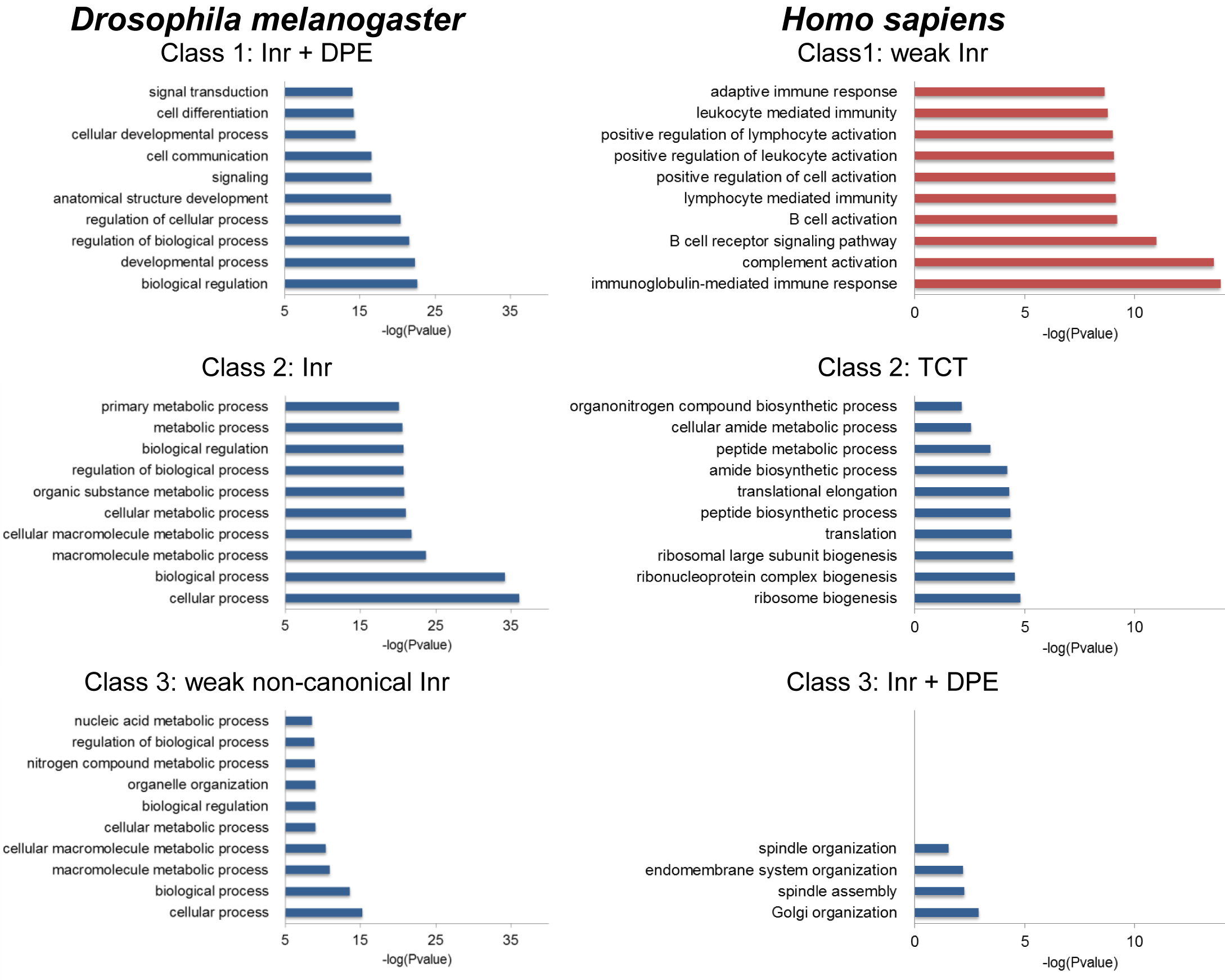

Supplement: S4 Fig — Gene lists comprising the Drosophila melanogaster classes identified by the basic probabilistic partitioning EM algorithm (Fig 2) and the human classes identified by the extended probabilistic partitioning EM algorithm (Fig 3), was analyzed using PANTHER-GO slim Biological Process annotation data set [45]. The enrichment scores are presented as -log10(P value). GO terms enrichment is indicated by blue bars, whereas GO terms depletion is indicated by red bars. The Inr-containing Drosophila Class 2 and the weak non-canonical Inr motif-containing Class 3 are enriched for metabolic and biological processes. Class 1 of human promoters identified using the new extended algorithm is depleted for immune response. As promoters in this class account for 87.9% of promoters of the human promoters, the significance of this depletion is unclear. Numerical data are provided in S2 File. (TIF) [file pcbi.1009256.s009.tif]

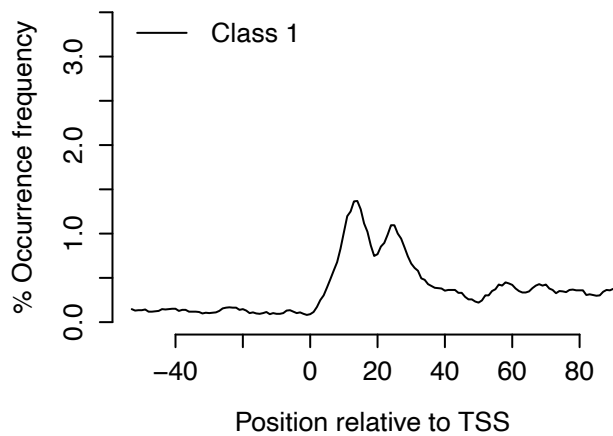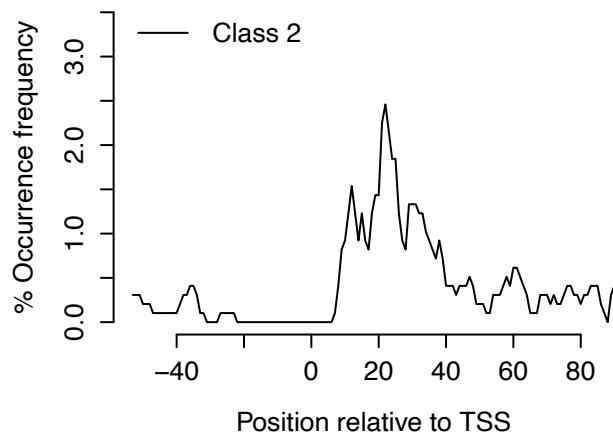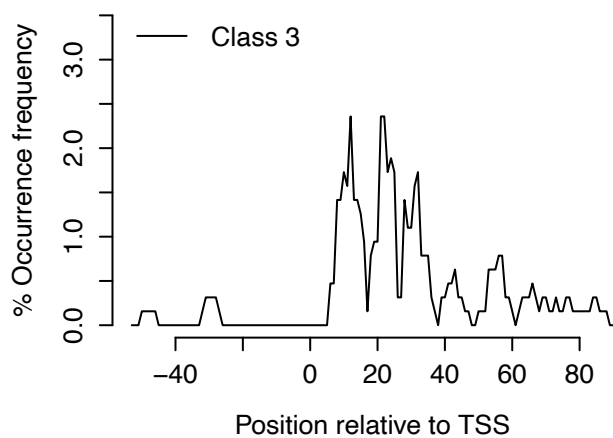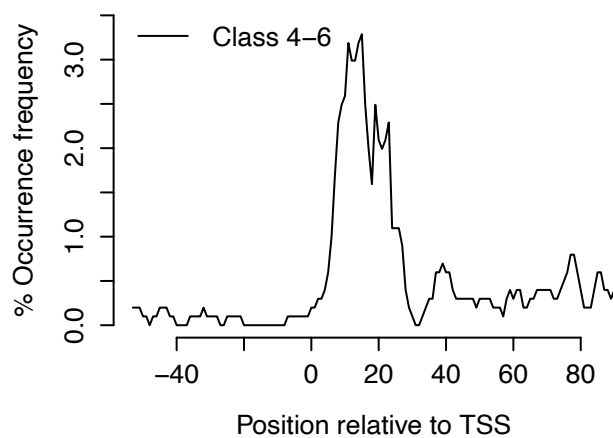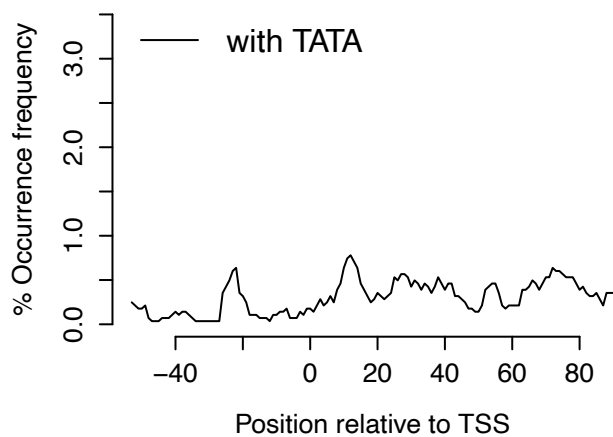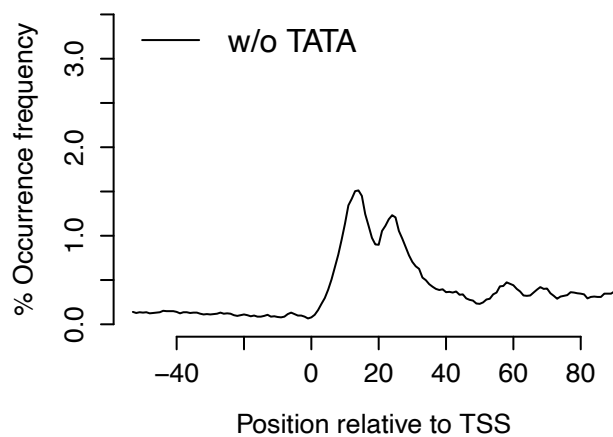

Supplement: S5 Fig — The motif enrichment profiles were generated with the TF binding site matrix MA0095.2 YY1 (Length=12) from JASPAR 2020 [71] with a window size of 16 and in unidirectional search mode. Due to the small window size, the height of the peaks does not reflect the total fraction of promoters that contain a YY1 motif in the corresponding promoter class. Numerical data (provided in S2 File) were generated using the web interface of the OProf program from the Signal Search Analysis server [70]. (PDF) [file pcbi.1009256.s010.pdf]

LRCH4

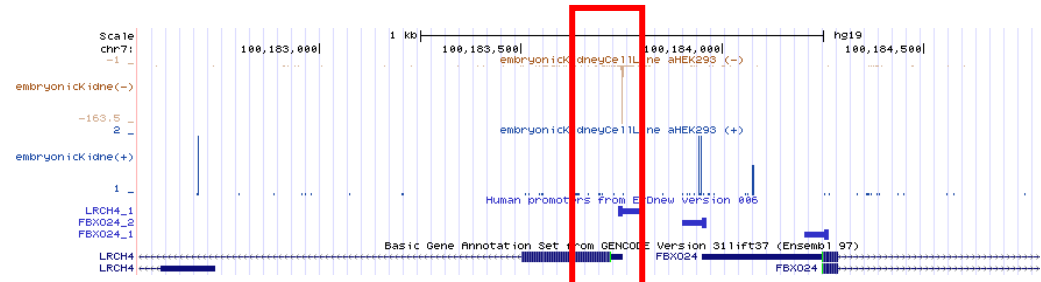

ANP32E

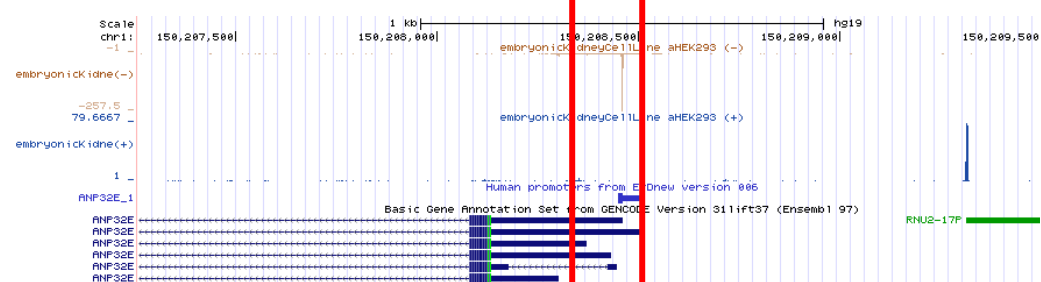

CKS2

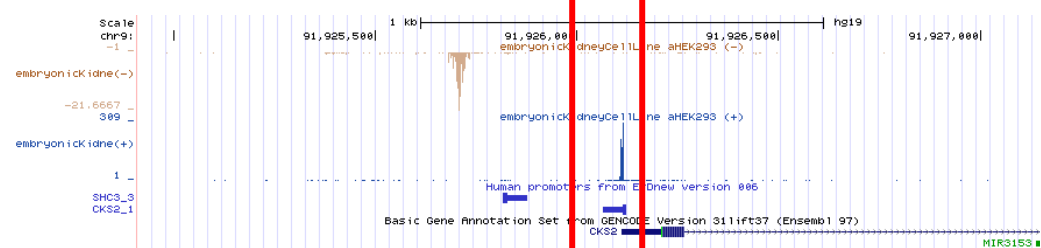

CELF1

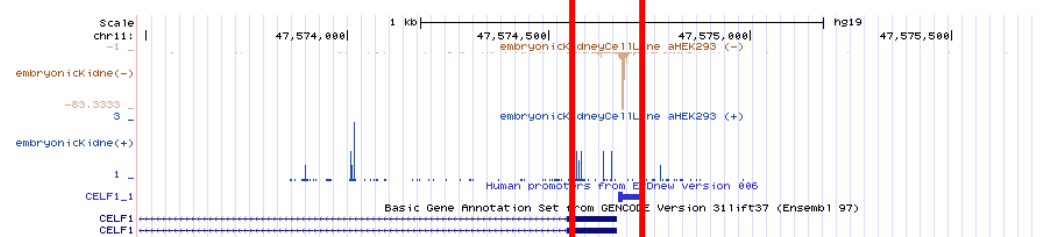

CTSA

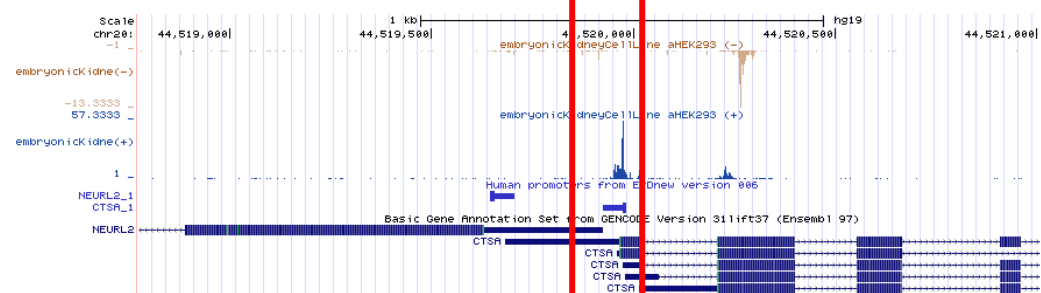

Supplement: S6 Fig — FANTOM5-generated CAGE tags distribution of individual promoters in HEK-293 cells was manually examined using the EPDnew viewer, in order to determine their transcription initiation pattern. (PDF) [file pcbi.1009256.s011.pdf]

A

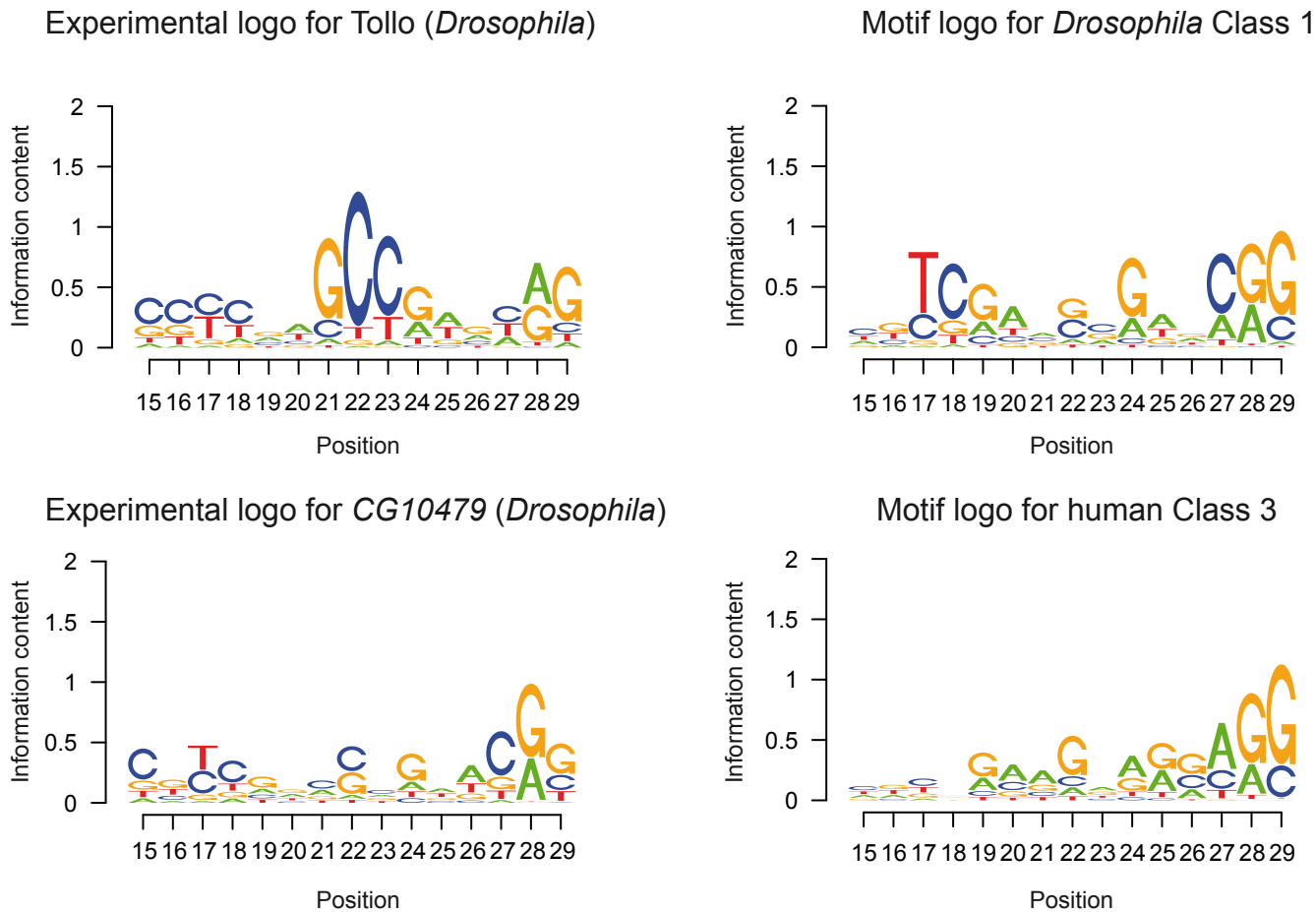

B

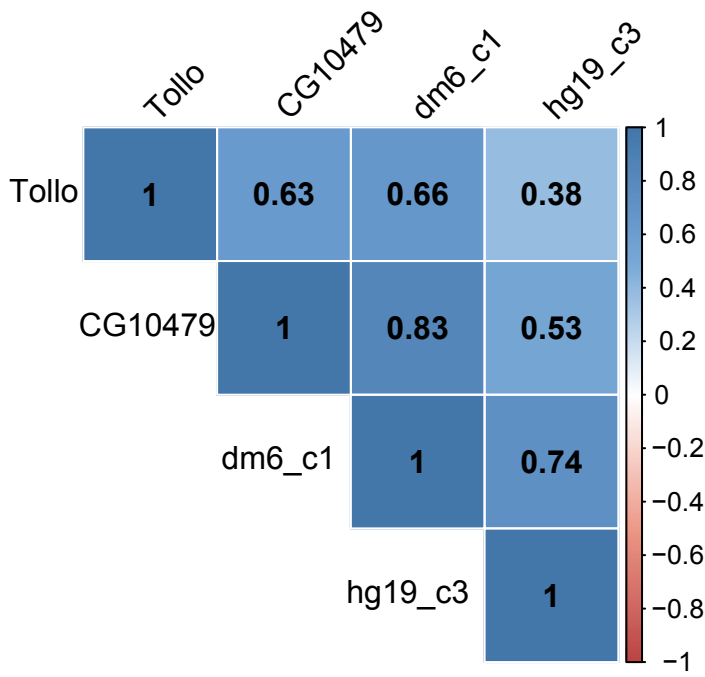

Supplement: S7 Fig — (A) Experimental logos are based on exhaustive single-base mutational analysis of the +15 to +29 region of two Drosophila promoters [22]. Relative expression values were rescaled such as to sum up to one at each position. The sequence motif logos were extracted from the logos shown in Figs 2 and 3. All logos have been over-skewed with an exponent of 2 to highlight differences between them. (B) Correlation plot showing Pearson correlation coefficients computed from the base probabilities underlying the logos. Note the high correlation of the CG10479 experimental logo with the Drosophila motif logo. Numerical data are provided in S2 File. (PDF) [file pcbi.1009256.s012.pdf]
