# Supplementary material for: Computational identification and experimental characterization of preferred downstream positions in human core promoters
Source: PLoS Comput Biol. 2021 Aug 12;17(8):e1009256. doi: 10.1371/journal.pcbi.1009256 (PMC8384218; doi:10.1371/journal.pcbi.1009256)
Supplement: Attachment — Submitted filename: Response to Reviewers Comments.pdf [file pcbi.1009256.s013.pdf]

We thank the reviewers for their valuable comments and insight. Their constructive suggestions have greatly helped in improving the manuscript, which now includes new experimental and computational analyses performed in response to reviewers' comments and suggestions.
The revised manuscript includes: 1 new figure depicting the analysis of nucleosome positioning, 3 new supplementary figures (depicting the enrichment of the *Drosophila* DPE motif logo in human promoters at the same downstream positions as in *Drosophila* promoters, GO terms analysis, and the positional distribution of the YY1 motif in the human promoter classes identified in this work), 2 revised figures (including reporter assays using 10 newly constructed plasmids, and ATAC-seq line plots analysis of different promoter classes), 1 new supplementary table, 1 revised table, 1 revised supplementary table, and textual revisions (*e.g.* clarification of promoter selection criteria).

Below please find a point-by-point response to all of the reviewers' comments. Line numbers refer to the revised manuscript with the tracked changes.

Reviewer #1:
We thank reviewer #1 for his/her constructive comments that helped to improve this manuscript. New data added according to his/her comments are presented in the **new S4 Fig, revised Table 1 and revised Figure 8. We have also revised Fig 4.**
Line numbers refer to the revised manuscript with the tracked changes.

Summary:
The authors investigated the structure of human core promoters with respect to the well-characterized Drosophila downstream promoter element (DPE), which is associated with an initiator element (Inr) located at a defined distance upstream from it. Based on some previous evidence, they hypothesized that DPE could exist in a fraction of human promoters and developed an original method of pattern discovery based on probabilistic partitioning algorithms by maximizing expectations which they adapted to extract patterns present in small sets of promoters. Among the discovered patterns, one looks similar to the Drosophila element Inr+DPE which they also exclusively found in other metazoan species. Using dual-luciferase assays, the authors showed a clear impact of DPE site mutations on promoter activity and a less obvious effect of spacer alteration. Overall, this study, which is nicely reported and pleasant to read, accessible and interesting for both bioinformatician and biologist communities, proposes a new well described methodology that could be generally applied when searching for complex motifs present in small but biologically relevant subsets of regions which are hardly detectable by classic methods. The functionality of the newly predicted human DPE is well supported by mutation assays, however, the need for strict spacing between Inr and DPE elements is less robust and would require further investigation.

Major issues:
1/ The authors selected 5 genes upon two scores, one from their motif, the second from a motif derived from a published experimental work on Drosophila. Those scores substantially vary

between the genes, for instance one of them has a negative score using their motif, which, to my understanding, would not represent a potential human Inr+DPE candidate. The authors should clarify the criteria applied for this selection.

We apologize for failing to describe the selection criteria applied. We revised the text accordingly and hope the revised text clarifies this issue.
In general, the ElemeNT score was used as an additional, auxiliary, criteria to choose from the EM algorithm-based list of potential targets. Since the experimental analysis and fine-tuning of the initial computational analysis were done in parallel, we used the well-characterized ElemeNT algorithm in order to detect the most promising candidates to be experimentally analyzed.

In addition, we have computed Z-scores by shuffling each promoter sequence 100 times, as log-odds scores are strongly influenced by base composition. These values are provided in the revised Table 1.
Indeed, the good agreement between the predicted EM and ElemeNT-based scores, as well as the experimental validation of the EM motif, indicates that "transcriptional regulation of human promoters via the downstream region is an important regulatory mechanism, likely conserved among metazoans".
The text now reads (lines 452-455 of the revised manuscript):
"To narrow down the selection towards experimental validation, we applied the ElemeNT algorithm (52) to detect possible initiator and DPE motif, based on PWMs constructed using experimental work in Drosophila (Sloutskin 2015, ref #52)."

For Fig. 7B, we started from a broader list of potentially-functional promoters and used the characteristics of LRCH4 as a reference, including the ElemeNT score, expression levels and initiation type. The criteria for selection of experimental candidates is described in lines 498-513 of the revised manuscript.
The text now states:
"Since the reduction in the LRCH4 reporter activity was more pronounced than that of the ANP32E gene (p-value 0.016) (as may have been expected based on the ElemeNT score, Table 1), we used the characteristics of LRCH4 as a reference. To this end, we started from a broader list of potentially-functional promoters. The resulting list was analyzed using ElemeNT, with the DPE score required to be >0.2 and accompanied by a Bridge element, similarly to LRCH4. The absence of a TATA-box was verified as well. We next applied a new additional cut-off expression levels criterion (based on HEK293 CAGE data generated by FANTOM5 consortium) - higher than or similar to those of LRCH4 - to select candidate promoters that would likely be expressed in our experimental system. Moreover, transcription initiation pattern (sharp or broad) was manually determined using the EPDnew website for each examined gene, based on the distribution of CAGE tags around the reported transcription start site.

2/ The authors evaluated the importance of the spacing between the Inr and the putative DPE in 2 of these genes by adding or deleting 2 nucleotides at 2 different positions, and reported a consistent decrease of activity for one of the genes, while the readout is less clear for the other which show higher variability between replicates and non significant decrease for the deletion at position 10 which makes the promoter activity of this gene not strictly relying on the spacing. The analysis of a third candidate may reinforce their conclusion.

To address this issue, we constructed mutant promoter constructs series for two additional candidate promoters, namely ANP32E and CELF1, and analyzed them in a similar manner.
In addition, the error bars used in the original submission for LRCH4 and CKS2 series were mistakenly representing the standard deviation (STDEV) instead of the standard error of the mean (SEM) of the experimental results. We apologize for this mistake, which has been corrected in the new version of the figure.

All the results are provided in the revised Figure 8. Insertion or deletion of 2 nucleotides at position +18 of each of the tested promoters, namely, LRCH4, ANP32E, CKS2 and CELF1, resulted in reduced activities. Moreover, insertion of 2 nucleotides at position +10 of each of these promoters, also resulted in reduced activities. Nevertheless, as deletion of 2 nucleotides at position +10 did not dramatically alter the activity of ANP32E, CKS2 and CELF1, we deleted the word: strict (in the context of strict spacing dependency) in the abstract and the text (lines 47, 527, 530 & 705).

The revised text now includes these results and potential explanations (lines 536-547):
"Although deletion of 2 nucleotides in position 10 of the ANP32E, CKS2 and CELF1 promoters did not result in reduced activity, significantly reduced activities were detected in CKS2 promoters for mutants in which 2 nucleotides were either deleted or added at position 18, and when 2 nucleotides were added in position 10. Interestingly, position +10 of DPE-containing promoters is located in a DNase I protected region (Juven-Gershon 2006, ref#14, Kutach 2000, ref #28), suggesting that this stretch contains a yet-uncharacterized sequence motif that could be independent of the Inr, and might contribute to the transcriptional activities. Therefore, the differences in the transcriptional output for position 10 mutants were expected to be less profound than for position 18 mutants. Indeed, deletion of 2 nucleotides at position +10 affected the reporter activities to a lesser extent compared to position +18. "

3/ In the discussion, three new results appear that were not present neither in the Results section nor in the methods.
- the first one concerns the functional enrichment of genes belonging to the motif classes 2 and 3 (l. 492, l. 553)

A new figure presenting the GO terms associated with each class, along with its p-values, is now included in the revised manuscript (S4 Fig). The Results section now includes the following text (lines 266-277):

“In addition, we performed GO terms analysis on the Drosophila melanogaster classes identified by the basic probabilistic partitioning EM algorithm (Fig 2) and the Homo sapiens classes identified by the extended probabilistic partitioning EM algorithm (Fig 3) using PANTHER (Mi 2019, ref#46). The canonical Inr and DPE motif-containing Drosophila Class 1 was found to be enriched for the regulation of biological processes and developmental processes (S4 Fig), as expected (Juven-Gershon 2008, ref#25, Zehavi 2014, ref#26). The GO term analysis of human Class 2, TCT enriched promoters, confirmed the enrichment of ribosomal and protein synthesis-related genes, as previously demonstrated (Parry 2010, ref#45). The Inr+DPE motif-containing human Class 3 genes were found to be enriched for spindle assembly and organization, yet less significant. No GO terms enrichment was detected for either of the human classes 4 to 6, exhibiting the (GCN)n pattern in three different frames.”

- the second reports a comparison of discovered Inr+DPE motifs in human and Drosophila with experimentally derived MTE motifs from 2 Drosophila genes (l. 509-531)

As this figure represents the analysis of previously published results, we feel it best fits in the discussion section. We moved the figure to the supplementary materials and it is now S7 Fig.

- the third corresponds to the ATG phasing with the 3 motif classes showing CGN repeats (l. 570).

We moved S1 Table to the results section (lines 225-227), as requested. The revised manuscript now includes the following text:
“*The 3 bp periodicity suggests a function in translation. Indeed, this hypothesis is further supported by the fact the trinucleotide repeats are preferentially preceded by in-frame translation initiation codons in all three classes (S1 Table).”*

4/ The authors applied the basic EM algorithm on eight eukaryotes species, and found an exclusive presence of Inr+DPE motifs only in metazoan. However, the use of the extended algorithm could have highlighted minor motifs in non-metazoan species as it allowed to detect Inr+DPE motifs in human.

The focus in this study was the long-standing question of whether there is a human downstream element that resembles the well-characterized *Drosophila* DPE. We believe the new algorithm has the potential to identify new motifs in other biological contexts as well, and would encourage anyone interested in the analysis of additional organisms to use the code made available via github to explore their organism of choice.

Minor issue:

The ATAC-seq profiles are not easy to interpret and would gain in clarity when coming to comparison by joining the profiles in one plot either by species or all together (using line plots for instance) enlarging by this way the x-axis which would help to spot the precise positions which are the main points of the graphs.

ATAC-seq line plots have been generated and are now represented in the revised Figure 4.

Reviewer #2:

We thank reviewer #2 for his/her constructive comments that helped to improve this manuscript. New data added according to his/her comments are presented in the **new Fig 5, new S1 Fig, new S4 Fig, new S5 Fig, and new S2 Table. We have also revised Fig 4.** Line numbers refer to the revised manuscript with the tracked changes.

The manuscript by Dreos et al describe a computational discovery and experimental validation of long-elusive mammalian equivalent of the Drosophila downstream promoter element (DPE) in human promoters.

The known features of Drosophila DPE and the promoters that contain it are:

- It is present at nearly 30% of the promoters of protein-coding genes.
- It is highly overrepresented in genes associated with development and developmental gene regulation.
- It is associated with focused promoters, with a fixed (to a single bp) spacing relative to their preferred transcription start site.

On the other hand, the human DPE that the authors report has the following features:

- It is present at around 3% of promoters of protein-coding genes. While the promoters that contain it have a better-than average defined Inr, the DPE motif itself is weak enough (ca 2 bits of information) that it can probably be found at more than 3% of arbitrary positions within the promoter.

The analysis of DPE element distribution around all *D. melanogaster* and human promoters is presented in the new S1 Fig. Strikingly, we have detected a clear enrichment at the same downstream positions as for *Drosophila* promoters, indicating it is rather a functional element.

The revised text now states (lines 171-175):

"Strikingly, scanning human promoters using the DPE motif logo revealed a clear enrichment at the same downstream positions as in Drosophila promoters (S1 Fig). This result indicates that a downstream motif, which is similar to the Drosophila DPE, might be conserved to human promoters and potentially reflect its functional importance."

- The genes with DPE-containing promoters show no association with development or gene regulation: the authors mention this in passing in Discussion, without showing analysis to support this (admittedly negative) observation.

A new figure presenting the GO terms associated with each class, along with its p-values, is now included in the revised manuscript (S4 Fig). The Results section now includes the following text (lines 266-277):

“In addition, we performed GO terms analysis on the Drosophila melanogaster classes identified by the basic probabilistic partitioning EM algorithm (Fig 2) and the Homo sapiens classes identified by the extended probabilistic partitioning EM algorithm (Fig 3) using PANTHER (Mi 2019, ref#46). The canonical Inr and DPE motif-containing Drosophila Class 1 was found to be enriched for the regulation of biological processes and developmental processes (S4 Fig), as expected (Juven-Gershon 2008, ref#25, Zehavi 2014, ref#26). The GO term analysis of human Class 2, TCT enriched promoters, confirmed the enrichment of ribosomal and protein synthesis-related genes, as previously demonstrated (Parry 2010, ref#45). The Inr+DPE motif-containing human Class 3 genes were found to be enriched for spindle assembly and organization, yet less significant. No GO terms enrichment was detected for either of the human classes 4 to 6, exhibiting the (GCN)n pattern in three different frames.”

- It is unclear whether this set of promoters is mostly focused (no such analysis is presented), although the authors hand-pick focused candidates for experimental validation.

The new Fig 5 panels A and E now analyze the CAGE distribution around the promoters of different classes. Moreover, the distribution of promoter classes with “shape” status (narrow/broad) is included in the new S2 Table.
The revised text includes a section entitled: Association of human Inr+DPE promoters with other genomic features”, including the following sentence (lines 339-341):
“Do their less frequent human counterparts show the same trends? Indeed, human Inr+DPE promoters are more focused than other promoters of other classes (Fig 5 and S2 Table).”

Given the significant differences, the authors dwell surprisingly little on the causes for them. In Drosophila, Inr+DPE promoters are a well-defined functional and architectural class associated with genes responsive to multiple enhancers, including those at long distances from the promoter (as shown by Stark lab STARR-seq papers).

The following sentence was added to the discussion (lines 676-679):
“Interestingly, STARR-seq analysis demonstrated that Drosophila Inr+DPE promoters are associated with a distinct class of enhancers (Zabidi 2015, ref#15). The association of human Inr+DPE promoters with a specific subset of enhancers remains to be determined.”

This has either been acquired since the last common ancestor of insects and vertebrates, or it was an ancestral state that has subsequently been largely supplanted by other promoter type in vertebrates, leaving only a small minority of promoters with DPE-dependent architecture. Either that, or the mammalian DPE, while superficially similar to the Drosophila one, is something different altogether. (The results in the recent paper from Kadonaga lab, while stronger on experimental validation, are also amenable to the same questions.)

The ATAC-seq profiles of the Inr+DPE promoter classes from human and *Drosophila* (revised Fig 4) are not only highly structured, but also similar to each other, in particular in the proximal downstream region, indicating that the human and *Drosophila* Inr+DPE promoter classes are homologous in biochemical terms, *i.e.* they interact with a homologous protein complex. Moreover, following the specific comments of reviewer #2 (below) we examined the nucleosome distribution around *Homo sapiens* and *Drosophila melanogaster* TSSs and discovered that Inr promoters show an open chromatin configuration in both organisms, whereas TATA-box and Inr-DPE promoters demonstrate a closer conformation (new Fig 5). Additionally, for both organisms, a strong 10bp periodic signal present in Inr promoters, is strongly reduced by the presence of the Inr-DPE or the TATA-box. Taken together, the DPE is likely an ancestral feature, and not "something different altogether". However, it may have functionally diverged (lines 355-357):
" While they are structurally similar, and (based on ATAC-seq footprints) most likely engage in direct contact with homologous proteins, they may have functionally diversified in the vertebrate and insect lineages."
and (lines 673-676):
"Our analysis shows that the Drosophila and human DPE are similar, but not identical in all respects. While the Drosophila DPE is a robust driver of gene expression, the identified preferred positions among human promoters, although important, are probably used for "fine-tuning" of transcriptional activity."

Specific comments:
- Drosophila DPE promoters, like TATA promoters in all Metazoa so far, are motif-dependent and, unlike dispersed promoters, do not feature a stably positioned downstream nucleosome. How does the human DPE promoter class look in that regard? Does it a) have a strong nucleosome position, which would argue for nucleosome-dependent transcriptional initiation, another major difference from the Drosophila DPE, b) no strong nucleosome position, which would lead one to expect that it is always associated with focused transcription start site or 3) does the nucleosome position depend, even within this class, on whether transcription initiation is focused or dispersed?

We thank the reviewer for pointing this important aspect. Analysis of nucleosome positioning is now presented in the new Fig 5. The distribution of promoter classes with "shape" status (narrow/broad) is included in the new S2 Table. Overall, we find reduced nucleosome positioning signals in human Inr+DPE promoters as compared to the major human promoter class.
The results are described in the Results in a section entitled "*Association of human Inr+DPE promoters with other genomic features*" (lines 336-382).

" In Drosophila, promoters with an Inr+DPE element constitute an abundant class associated with specific architectural and functional properties, including focused

transcription initiation, a disordered nucleosome organization and over-representation in developmental genes (Danino 2015, ref#1, Lenhard 2012, ref#3). Do their less frequent human counterparts show the same trends? Indeed, human Inr+DPE promoters are more focused than other promoters of other classes (Fig 5 and S2 Table). With regard to nucleosome architecture, nucleosome occupancy profiles derived from human MNase data (Fig 5, panels B,F) are not conclusive due their noisiness (presumably related to the small number of promoters in this class). We tried to circumvent this limitation by quantifying the sequence-intrinsic nucleosome positioning signal as a proxy (Fig 5, panels C,G) for experimentally determined nucleosome architecture. To this end, we relied on a previously published method based on spectral analysis of dinucleotode periodicity (Dreos 2016, ref #10). Since TATA-box containing promoters were also reported to have disorganized nucleosomes, we included this class as a control. The results presented in Fig 5 show a reduced but not completely absent nucleosome positioning signal in human Inr+DPE promoters. The peak at period 10 is substantially lower than in the Inr-only classes from both species, but definitely not absent as in the Drosophila Inr+DPE promoter class. In summary, human Inr+DPE promoters only partially reproduce characteristic genomic features associated with their counterparts in Drosophila. While they are structurally similar, and (based on ATAC-seq footprints) most likely engage in direct contact with homologous proteins, they may have functionally diversified in the vertebrate and insect lineages…
It is possible that nucleosome positioning, among other molecular mechanisms, contributes to the transcriptional output of DPE-containing promoters. Nevertheless, our observations indicate that the DPE sequence motif affects transcriptional activity of DPE-dependent promoters."

- In case of stable nucleosome position, could the validation experiments that remove the most informative DPE motif positions or change the spacing between DPE and TSS be interpreted via their effect on the nucleosome positioning? For example, if a nucleosome is shifted 2bp left or right, the optimal position of the Inr would shift with it, and if there is no optimal Inr motif 2bp left or right, the efficiency of initiation would decrease?
The problem with such functional probing is that there is no guarantee that the changes authors introduce have anything to do with the DPE motif itself, or that they wouldn't give similar results on other promoters with similar TSS dispersion and nucleosome positioning properties. That also holds for the three human promoters apparently historically demonstrated to have functional DPE (IRF1, CALM2, TAF7).

The reviewer's criticism is applicable to any promoter mutagenesis experiment using reporter assays in transfected cells. It is, of course, possible that altered nucleosome positioning and a great variety of other potentially impaired molecular interactions contribute to the reduced transcriptional activity observed with our mutant promoters. However, the consistent down-trend observed with four different promoters suggests that the majority of our rationally designed mutants do indeed reduce transcription through a DPE-mediated mechanism.

- The decreases in promoter activity upon mutating away the proposed DPE motif are relatively mild. If it is an essential part of the architecture of those promoters like TATA (as opposed to e.g. providing some gentle cues), shouldn't the effect be much more dramatic? Doesn't the abolishment of DPE in Drosophila developmental promoters have a much more pronounced effect?

As discussed above, the *Drosophila* and human DPE cannot be directly compared. While the *Drosophila* DPE is a robust driver of gene expression, the identified preferred positions among human promoters *"although important, are probably used for "fine-tuning" of transcriptional activity."* (lines 675-676 of the revised manuscript). Moreover, when testing for *Drosophila* DPE activity, a mutation of either 4 of 7 nucleotides is commonly used (Kutach et al 2000 (PMID: 10848601), Lim et al 2004 (PMID: 15231738), Zehavi et al 2014 (PMID: 24634215), Shir-Shapira et al 2019 (PMID: 30998799), and more). Notably, we tested a mutation of only 3 nucleotides - at positions +24, +28 and +29. The effect of these 3 nucleotides alone, even if considered mild, together with the results of the shifting experiments, supports the contribution of these downstream positions to transcriptional activity.

- Partitioning of Drosophila and human promoters using basic (Fig 2) and human promoters using new extended EM algorithm (Fig 3): The obtained classes should be checked for association with other promoter elements outside the (-10,+40) region, to see how they correspond to previously proposed promoter types (which ones have TATA, which ones have nucleosome positioning signal, etc.).

Based on the analysis described in the new Fig 5 and S2 table, Human Inr-DPE promoters have both a reduced TATA-box frequency and a reduced nucleosome positioning signal in the downstream region.

Also, the position of proximal downstream YY1 motif should be of interest - it is present at about the same position, has some similarity to the discovered DPE motif, and clips the extent of the dispersed TSS. An older paper (https://genome.cshlp.org/content/17/6/798) has found both YY1 and the novel "motif8" element at approximately the same position

The analysis of YY1 motif enrichment profiles of different human promoter classes is now included in the new S5 Fig. We were unable to find Motif 8 in a numerical form. The revised text includes the following (lines 364-378):

"An earlier paper (Xi 2007, ref#49) reported strong over-representation of the YY1 motif in the proximal downstream region of human promoters, approximately at the same location where the DPE element occurs. We thus analyzed the positional distribution of the YY1 motif in the human promoter classes identified in this work (S5 Fig). Here, the TATA-box containing (TATA+) and Inr+DPE promoters show contrasting trends. While the YY1 motif is virtually absent in TATA+ promoters, it is over-represented in all specialized minor promoter classes (TCT, Inr+DPE, poly-GCN 1,2,3). The motif occurrence profiles show 2-3 peaks separated by about 10 bp, indicative of one-sided binding to the double helix. Interestingly, the Inr+DPE class

shows the clearest 10-bp periodicity, suggesting that promoters of this class are tightly bound by a large protein complex on the other side of the DNA. This scenario would be entirely compatible with the prevalent view that Inr-DPE promoters make direct DNA-protein contacts with components of the TFIID complex. In summary, our motif co-occurrence analysis revealed additional distinctive features of human Inr+DPE promoters, further justifying their status as a separate promoter class."

- The ATAC-seq footprints in Fig 4 look strange - I do not understand how "single-base resolution" footprints are obtained - was it just by taking the midpoints of the fragments? In such case a "V-plot" would be more interesting, as it would reveal the positions of nucleotide-size fragments and help answer at least some of the above questions about nucleosome positioning in different classes.

ATAC-seq line plots have been generated, along with a more detailed description. The modified graphs are now part of the new Figure 4. We also added new text, including a new reference (Karabacak Calviello et al. 2019) to better explain the technique and rationale behind digital ATAC-seq foot-printing (lines 292-298):

"One way to approach this question is by looking at chromatin accessibility footprints (Hesselberth 2009, ref#47). Both DNase-seq and ATAC-seq assays provide genome-wide maps of DNA accessibility at single base resolution. The binding of a protein complex to a genomic region changes the exposure of individual phosphodiester bonds to DNase and transposase in the bound region. A characteristic footprint is revealed if sparse data from many DNA regions are super-imposed in an aggregation plot (as in (Karabacak Calviello 2019, ref#48))."

- Regarding the super-core promoter: is there any evidence that TATA box and DPE in it are used simultaneously instead of being mutually exclusive (i.e. either one or the other is used in a particular transcription initiation event but not both)?

The super core promoter seems to rely both on the TATA box and the DPE, as mutating either of these elements reduce its transcriptional activity (Juven-Gershon 2006, ref#14). However, analysis of individual initiation events is not available.

Reviewer #3:

We thank reviewer #3 for his/her constructive comments that helped to improve this manuscript. **Fig 4 and Table 1 were revised according to his/her comments.**

Line numbers refer to the revised manuscript with the tracked changes.

In this paper Dreos, et al, develop a method to identify patterns in core promoters using a modified expectation maximization algorithm (EM). The method enables the identification of hidden patterns that have been profoundly characterized in Drosophila but that have escaped clear determination in human promoters.

Core promoters are defined as the -40 +40 regions around transcriptional start sites, these regions are bound by the RNA polymerase machinery and while clear patterns of sequences

required for this function have been characterized in Drosophila, their characterization in other organisms has been challenging.
Authors apply a probabilistic partition algorithm based on expectation maximization to enable discovery of highly variable but conserved sequences in core promoters in human and Drosophila. Authors recapitulate previously known biologically meaningful patterns in the Drosophila data set and are able to discover previously known and new biology in human promoters. Particularly interesting, the new insight in patterns in human promoters are similar to patterns in Drosophila. Moreover, the results of the program in other organisms reveals a conservation pattern across species.
Promoter predictions are then experimentally tested and the bases that were predicted to be more relevant in the promoter function are the ones that when mutated have a higher impact on the promoter function.

2) Specific comments for revision:
a) Major.
The EM algorithm is shown to be able to recover relevant motifs in the promoters, however, for the selection of promoters to be tested experimentally authors use the tool ElemeNT with the motifs provided by that tool, instead of using the motifs discovered by EM. For this reviewer is not clear why this decision was made, I understand the tools ElemeNT has been implemented to run pattern-matching looking for positional preferences, but I would expect the usage of the motifs discovered and described in this article and not others. Is important that this is clarified in the text.

We apologize for failing to describe the selection criteria applied. In response to this comment (made by both reviewers #1 and #3), we revised the text accordingly and hope the revised text clarifies this issue.
In general, the ElemeNT score was used as an additional, auxiliary, criteria to choose from the EM algorithm-based list of potential targets. Since the experimental analysis and fine-tuning of the initial computational analysis were done in parallel, we used the well-characterized ElemeNT algorithm in order to detect the most promising candidates to be experimentally analyzed.
The text now reads (lines 452-455 of the revised manuscript):
"To narrow down the selection towards experimental validation, we applied the ElemeNT algorithm (52) to detect possible initiator and DPE motif, based on PWMs constructed using experimental work in Drosophila (Sloutskin 2015, ref #52)."

For Fig. 7B, we started from a broader list of potentially-functional promoters and used the characteristics of LRCH4 as a reference, including the ElemeNT score, expression levels and initiation type. The criteria for selection of experimental candidates is described in lines 498-513 of the revised manuscript.
The text now states:
"Since the reduction in the LRCH4 reporter activity was more pronounced than that of the ANP32E gene (p-value 0.016) (as may have been expected based on the ElemeNT score, Table 1), we used the characteristics of LRCH4 as a reference. To

this end, we started from a broader list of potentially-functional promoters. The resulting list was analyzed using ElemeNT, with the DPE score required to be >0.2 and accompanied by a Bridge element, similarly to LRCH4. The absence of a TATA-box was verified as well. We next applied a new additional cut-off expression levels criterion (based on HEK293 CAGE data generated by FANTOM5 consortium) - higher than or similar to those of LRCH4 - to select candidate promoters that would likely be expressed in our experimental system. Moreover, transcription initiation pattern (sharp or broad) was manually determined using the EPDnew website for each examined gene, based on the distribution of CAGE tags around the reported transcription start site.

b) Minor

Figure 5A: Maintain negative positions as reference to the previous figure4.

ATAC-seq line plots have been generated. The modified graphs are now part of the new Figure 4.

Line 258 should be revised.

We thank the reviewer for pointing out this typo. The text now reads: *"The **question** whether…"*
