# Supplementary material for: Computational identification and experimental characterization of preferred downstream positions in human core promoters
Source: PLoS Comput Biol. 2021 Aug 12;17(8):e1009256. doi: 10.1371/journal.pcbi.1009256 (PMC8384218; doi:10.1371/journal.pcbi.1009256)
Supplement: Attachment — Submitted filename: Response to Reviewers Comments_Round 2.pdf [file pcbi.1009256.s014.pdf]

We thank the reviewers for their valuable comments and suggestions that have helped in improving the manuscript.
Below please find a point-by-point response to all of the reviewers' comments. Line numbers refer to the revised manuscript with the tracked changes.

**Reviewer #1:**
The authors seriously considered our previous remarks and largely assessed the highlighted issues. This significantly improved the paper. However, the score definitions (log-odds and z-scores) and its use remain somehow unclear to me.
The authors explain that the log scores are strongly influenced by base composition, so can we rely on this score? Is the z-score a better estimation than the log-odd then? If a user was about to use the EM algorithm on its favorite species and want to test some of its hits, what the authors would recommend? I may have missed the information but a clear definition of the scores, their potential bias and how to interpret them could help future users.

A new paragraph describing the computation and definition of log-odds and Z-scores, as well as the difference between them, are now included in the Methods section.
The text now reads (lines 761-775 of the revised manuscript):
***"Computation of log-odds and Z-scores***
*The probability matrix for the human promoter class 3 (Fig 3, numerical data in File S2) was used to compute "log-odds" and "Z-scores" for wild-type and mutant promoter sequences (Table 1 and S3 Table). Log-odds scores were computed by converting the probability matrix obtained from probabilistic partitioning into a weight matrix assuming a uniform base composition*

$$w_{bi} = ln\left(\frac{p_{bi}}{0.25}\right) \qquad (6)$$

*and then summing up the position-specific weights corresponding to a particular sequence. Z-scores were estimated by normalizing the log-odds scores with respect to the mean and standard deviation of the scores obtained from 100 shuffled versions of the same promoter sequence. Note that according to Berg and von Hippel (63), a log-odds score computed from observed base frequencies should be inversely proportional to the binding energy of the corresponding protein-DNA complex. Z-scores, on the other hand, serve to estimate the probability that a motif match found in a real sequence could be due to chance."*

**Reviewer #2:**
The revised version of the manuscript by Dreos et al. has addressed many of my original concerns, and added a number of new analyses that resulted in interesting observations.
While the evidence still does not prove conclusively that the human Inr+DPE promoter shares the same evolutionary origin as the Drosophila one (3% vs 30% genes that contain it, and no clear functional preference vs clear developmental specialisation are

very important differences still), it provides a lot of observations for future study. I am especially excited about the differences in YY1 binding across classes.

We are also excited about the differences in YY1 binding across promoter classes. Thank you.

**Reviewer #3:**
I consider the authors have addressed all concerns and substantially improved their article.
I just noticed that they forgot to add the methods for the YY1 analysis that was included during revisions, please include them so the manuscript can be completed.

We apologize for that. A new paragraph describing the analysis of the YY1 motif enrichment is now included in the Methods section.
The text now reads (lines 808-813 of the revised manuscript):
***"YY1 motif enrichment profiles of different human promoter classes***
*The motif enrichment profiles were generated with the TF binding site matrix MA0095.2 YY1 (Length=12) from JASPAR 2020 (71) with a window size of 16 and in unidirectional search mode. Numerical data (provided in S2 File) were generated using the web interface of the OProf program from the Signal Search Analysis server (70)."*
